# Dr. West’s Case of Malformation of the Heart and Great Vessels, Attended with Cyanosis; with Remarks upon This Affection

**Published:** 1842-11-05

**Authors:** 


### MEDICAL EXAMINER.

NEW SERIES.

No. 45.] PHILADELPHIA, NOVEMBER 5, 1842. [Vol. I

TRANSACTIONS OF THE PATHOLOGICAL SOCIETY.

*Monday,* 31*st October.*

Dr. West read the report of

### *A Case of Malformation of the Heart and Great Vessels, attended with Cyanosis; with remarks upon this affection.*

The subject of this case was a boy, aged 8 years, of full size, well developed and possessing ordinary intelligence. His parents, who are remarkably robust and healthy, state he was born at the full time; that at birth his skin was slightly blue, but that this appearance, which they describe as resembling "the effect of cold," did not assume a permanent or marked character, so as to attract general attention, until after an attack of pertussis, which he had when about fifteen months old. From this period the discolouration became fixed and very intense, particularly about his face and hands. M. Louis mentions a case in which cyanosis was thus fully developed after pertussis. He had never suffered from palpitation of the heart or dyspnea, except after unusual exertion, nor had he ever been subject to lypothymia. His parents state that at times, when he was sitting erect and perfectly quiet, they have known his skin, for a few minutes, to lose entirely its characteristic dark hue. Such a fact would appear to be with difficulty reconcileable with the commonly received opinion of the nature of cyanosis, which attributes this affection to the simple transmission of black instead of red blood through the vessels, else why should this, which is always present in them, *ever* fail to impart its proper colour to the surface. If, on the contrary, we suppose that from some cause or other, situated at the heart, the blood is materially and permanently retarded in its course back to this organ, and that congestion, therefore, ensues throughout the whole venous system, and that of the skin in particular, why then, we can explain the varying shades of colour, as well as the entire disappearance at times of the cyanosis, upon the presumption that the blood thus temporarily has been enabled to pursue a comparatively unobstructed course, with consequent relief to the local congestion producing the cyanosis. Berard, in his article upon the Abnormal Anatomy of the Heart, in the Dict. de Medécine, thus argues against the doctrine that cyanosis is owing to the simple presence of black blood in the vessels. He can understand, he says, why certain parts to which the arterial blood habitually gives a vermillion tint, such as the lips and the cheeks, should assume a dark colour, when a mixed blood is in circulation through the vessels, even without any retardation of its course, but he is not able to see why the skin of the rest of the body, which is not sufficiently vascular to receive a *red* tinge from the arterial blood circulating through its vessels, should become *blue*, when this fluid happens to be changed in colour. It ought to remain white, he contends, in the one as in the other case, if the circulation be not modified. In reply to his own question, to what cause, therefore, are we to attribute the cyanosis? he says, "Evidently to the difficulty of the venous circulation and to the stasis of blood in this order of vessels." A positive fact upon this subject, stated by Breschet, seems to establish the point that something more than this mere presence of dark blood in the vessels is required to produce cyanosis. In that case the left subclavian arose from the pulmonary artery without the colour of the parts supplied by its ramifications being at all affected. Dr. Crampton has reported a case like our own in which the aorta was placed astride of the ventricles, unattended by cyanosis. As pertinent to this point we may quote the remark of Professor Fouquier, that the skin of the fœtus, in which nothing but black blood circulates, is nevertheless not cyanosed.

To return to the case before us. In July last this boy was suddenly seized with violent general convulsions, of several minutes duration. They had subsided when I saw him; he was then perfectly conscious, and complaining only of pain at the fore part of his head. He had slight dyspnea, but was able to assume a perfectly recumbent position in bed. The heart was beating tumultuously, with perfect confusion of all its sounds. The surface was every where intensely blue and cool; hands and feet quite cold. He was directed to be leeched to the head, with cold applications to it, and an injection to be given. On the next day he was able to be out of bed, and felt quite comfortably; although the cyanosis did not materially abate, he continued otherwise to improve until the third day from his first seizure. He was then again attacked in the same way with a convulsion, and almost immediately expired. The autopsy was made eighteen hours after death, with the assistance of my friends, Dr. Pepper and Mr. Wallace. The thorax only could be examined, from the limited time at our command.

The lungs, of full size, were congested throughout with very black blood; in other respects they were natural. The pericardium, which was remarkably prominent from the enlarged condition of the heart, contained a little yellow serum. The heart, in its substance, its coronary vessels, and in all its cavities, was remarkably turgid with dark blood. When emptied, it measured five inches in length, and seven in circumference, around the base of ventricles. Its cavities were all dilated and their walls thickened; those of the right side, however, in both these respects, much more so than the left ones. The actual capacity of the right was nearly double that of the left ones, showing, we think, that notwithstanding the community of the four cavities, by reason of the extensive opening between the ventricles presently to be described, those of the right side, nevertheless, preserved to a great extent their individuality, if we may so speak. These considerations strike us as important in contending for the operation of obstruction, at the right side of the heart, in producing cyanosis, where a communication exists between its opposite cavities.

The foramen ovale was closed. The opening existing between the ventricles and common to them and the aorta was circular, and about half an inch in diameter. It was situated at the posterior end of the base of the inter-ventricular septum, and did not interest the auriculo-ventricular orifice of either side. The aorta, the root of which was embraced within the limits of this opening, had an origin from each ventricle. Its valves were all perfect, and situated just at the mouth of the opening. The aorta measured an inch in diameter, with very firm parietes. The mitral valve overhung the opening described, at the left ventricle. The pulmonary artery presented some very peculiar deformities. The trunk of this vessel consisted of a conical fibrous looking projection, about half an inch high, and arising from the heart by a broad and spreading base. It entered at a somewhat acute angle the side of a straight vessel, two and a half lines in diameter, constituting its right and left branches, which were attached closely to the aorta and to the left auricle by condensed cellular substance. This projection, on being laid open, was found to contain a little papillary eminence, about one and a half lines high, arising from the heart. A small aperture at its apex, scarcely capable of admitting a large pin, was continuous with a passage through it, of the same dimensions, to the heart, where it opened at the superior left corner of the right ventricle. This aperture was almost concealed from view by its position, and was also rendered somewhat valvular, by the muscular columns of the cavity encroaching upon it laterally. Very little blood could possibly have reached the lung through it; their main supply, on the contrary, was furnished by the ductus arteriosus, which was still open. Within the pulmonary artery, at the point where this duct commenced, there existed a kind of valvular arrangement, indicative of the important part played by it in the circulation of the blood. This duct passed directly across from the aorta to the pulmonary artery, which latter vessel being perfectly straight where the duct joined it, necessarily occasioned the blood which reached it, to branch off into two directly opposite currents. From this whole arrangement, we can readily conceive how much the course of the blood from the right ventricle into the lungs, must have been retarded, the whole of it, nearly, having to pass at several right angles, from the relative position of the different vessels. The consequence of all this must necessarily have been considerable delay of the blood in the right cavities of the heart, producing their dilatation, and also preventing the free admission of blood into them, together with congestion of the whole venous system, and, as a matter of course, that of the skin, more particularly, where it exhibited itself in the character of cyanosis.

The pulmonary veins were only two in number, and smaller than natural; the venæ cavæ, which were very large, instead of entering the heart separately, were first united into a common trunk, which joined the auricle about its middle, and exactly at a right angle; a further mechanical impediment was here offered to the free return of blood to the heart; a salutary provision, doubtless, by which the force of the blood in this direction was brought into harmony with that which marked its embarrassed departure from the heart. Such a relationship must have prevented the disturbing consequences which would otherwise have attended the arrival of blood at the heart more rapidly than it could be expelled from it. This arrangement beautifully illustrates that law of compensation so frequently observable in the animal economy, and to the operation of which we are inclined to attribute the comparative freedom which this patient enjoyed from the usual consequences of so extensive a malformation of the heart and great vessels.

We have now finished the description of the specimen before the Society, which we were induced to present to it, chiefly because its pathological or anomalous characters illustrated so perfectly those congestive views of the nature of cyanosis, which seem to be most consistent with physiological and pathological analogy.

Morgagni, in relating a case of cyanosis, from communication of the right and left cavities, with contraction, also, of the orifice of the pulmonary artery, remarks that, in order to account for the livid colour in question, we must consider the influence of such contraction, (dependant, in his case, upon ossification, believed by him to be congenital) in producing embarrassment of the circulation of the black blood, whereby this must remain stagnant in the right ventricle, in the corresponding auricle, and of consequence, in the whole venous system, " whence," he says, " results the livid colour of the skin." Louis, in his memoir upon this affection, asserts that in more than half the cases, where cyanosis followed the communication of the right and left cavities, there was found contraction of the orifice of the pulmonary artery, conjoined with the above condition of the heart. The venous congestion, however, which is maintained by Louis and many others to be the essential character of cyanosis, in these cases of malformation of the heart, attended by the mixture of the two bloods, will of course be promoted by the inactivity of the capillaries themselves, super-induced by the presence, in them, of an imperfectly vitalized blood; the influence, therefore, of the latter circumstance is by no means to be overlooked or undervalued, in our inquiries into the mode of production of cyanosis. In opposition to the views to which we have called attention, and in favour of those more commonly received, it will doubtless be asked, how happens it then that cyanosis is rarely, if ever, observed, except where a direct communication exists between the opposite cavities of the heart, even though obstruction at its orifices should exist to the greatest extent? This question or objection might fairly be answered, it appears to us, by inquiring in return, why it is that cases now and then occur, in which, after death, extensive communications of this kind have been discovered, without the subjects of them during life having presented the smallest appearance of cyanosis? But the coincidence of these two lesions of the heart is almost a necessary one, since the diversion of the blood through the ductus arteriosus from the aorta into the lungs, must unavoidably produce a more or less perfect closure of the pulmonary orifice, by reason of its proper function being suspended. We cannot, of course, imagine the long continued independent existence of such extreme contraction of the pulmonary artery, amounting almost to obliteration, as would be necessary to the production of permanent cyanosis, since such a lesion would be incompatible with the existence of life; but when this is associated with an opening in the septum of the heart, by which the blood is able to find its way, although very indirectly and obstructedly, as we have seen, to the lungs, the difficulty just named is removed; still, however, even under these circumstances, the circulation is far from being easily and perfectly performed, and the train of consequences before alluded to, necessarily follows, and cyanosis is produced. In regard to the influence of the contraction of the pulmonary orifice, and in further corroboration of the views above expressed, it may be asked, what symptom is more common in cases of contraction of this, or any of the orifices at the right side of the heart, whether arising from actual disease in them, or from vegetations, and other affections of their valves, than the occurrence of lividity of the face, neck and other parts of the body? and what is this lividity, but an imperfect, limited cyanosis, which doubtless would become complete and general, but for the reasons already alluded to, when speaking of contraction of the pulmonary orifice?

In conversing upon this subject with our friend Dr. Hallowell, a member of this Society, who has paid particular attention to cardiac diseases, he remarked to us, that in the case of a patient who had died under his care with vegetations of the pulmonary valves, which had considerably occluded this opening, he had observed, just before death, the supervention of nearly complete cyanosis. We have referred, for confirmation of the congestive view of the nature of cyanosis, to physiological analogy and pathological experience.

We would therefore ask, what causes the dark, cyanosed appearance of a part, upon which strangulation has been exerted with sufficient force to arrest the venous, but not materially to interfere with the arterial circulation? Is it anything else than venous obstruction? What is it, again, that causes the duskiness or deep livid hue of the countenance, so commonly present in severe and extensive congestion of the lungs? It certainly is not for a moment supposed, that in such cases the blueness is produced by the heart circulating black blood, however fully we may admit the fact of the lungs being disabled under such circumstances, from properly discharging their decarbonizing functions. Congestion of the superficial capillaries from obstructed circulation through the lungs, is perfectly understood to be the whole cause of this appearance. Here we assume a point of obstruction, different to be sure from the one in cases of malformed heart with cyanosis, but nevertheless operative in precisely the same way, viz., by retarding the course of the blood. What is it, too, which mainly causes the dark colour of the skin in asphyxia; or even the blueness of the lips, of the ends of the fingers, and of other parts, in the chill of a common intermittent fever?

These, and all analogous cases, are but instances of partial cyanosis; and yet we seek no other explanation of them than the simple and satisfactory one of venous obstruction and congestion, originating in the latter case, probably at some of the great internal organs, and in the former from the same immediate cause, though induced, perhaps, by many different agencies. If, therefore, as we have seen in the instances just enumerated, some transient obstruction, wherever situated, is able to produce what may be considered a local cyanosis, why should not a general affection of this kind be expected from obstruction or obliteration of some of the great orifices at the heart, the very centre of circulation; particularly when we recollect that under such circumstances the venous system must be everywhere engorged and overloaded with blood, and its capacity of course exceedingly increased by the long continuance of such a condition.

Bertin, in combating the opinion that the simple mixture of the two bloods at the heart is the occasion of cyanosis, remarks that it is entirely inadmissible, since he has in his possession cases in which this affection did not exist, although the right and left hearts communicated, and others, where the cyanosis existed, although no communication of the kind was present. In fact, he says, if the *blue* colour of the skin be produced by this malformation, the same colour should be met with in every other part, which is contrary to observation. This want of universality of the blueness of all parts of the body in cyanosis, is a very strong argument against the common theory of its production. We do not even find the whole surface of the body discolored in this affection; the blueness being conspicuous only in certain parts. These remarks might be much extended; what we have said, however, will suffice to bring the question before the Society.

